# The Associations of Mental Health Disturbances, Self-Esteem, and Partner Violence Victimization with Condom Use in Spanish Adolescents

**DOI:** 10.3390/jcm11092467

**Published:** 2022-04-27

**Authors:** Miriam Sánchez-SanSegundo, Raquel Alarcó-Rosales, Ana Zaragoza-Martí, José A. Quesada-Rico, Eva Gabaldón-Bravo, José A. Hurtado-Sánchez

**Affiliations:** 1 Department of Health Psychology, Faculty of Health Science, University of Alicante, 03690 Alicante, Spain; miriam.sanchez@ua.es (M.S.-S.); alarco.rosales@gmail.com (R.A.-R.); 2 Department of Nursing, Faculty of Health Science, University of Alicante, 03690 Alicante, Spain; eva.gabaldon@ua.es (E.G.-B.); ja.hurtado@ua.es (J.A.H.-S.); 3 Department of Clinical Medicine, University of Miguel Hernández, 03202 Elche, Spain; jquesada@umh.es

**Keywords:** unprotected sex, self-esteem, adolescent, mental health, Spain

## Abstract

Risky sexual behaviors, such as not using a condom for preventing sexually transmittable diseases and unwanted pregnancies, are associated with emotional and psychological problems in adolescence, including higher levels of depression, anxiety, stress, and low self-esteem. Adolescents with a history of violence victimization are also at increased risk of engaging in sexual risk behaviors. In this study, we examined the associations between mental health disturbances and partner violence victimization and the non-use of condoms among young people in Spain. We also examined self-esteem as a mediator of mental health problems and unprotected sexual relationships. A cross-sectional study was conducted on a sample of 831 adolescents randomly selected from 25 high schools in Spain. From the initial sample, 285 students (144 men and 141 women) from high schools in Alicante (Spain) who reported having experienced sexual activity with at least one partner were approached. The results showed that one in three adolescents between 14 and 19 years old did not use condoms during sexual intercourse. The factors associated with the non-use of condoms in the estimated models of Poisson robust variance were having a history of physical, psychological, or sexual violence; having been expelled from school because of behavioral problems; and having higher levels of depression, anxiety, and stress and lower levels of self-esteem. Self-esteem was independently associated with the non-use of condoms. Having lower levels of self-esteem increased the risk of not using a condom during participants’ last sexual intercourse. These findings suggest the importance of developing public health strategies for mental health promotion to increase condom use among adolescents.

## 1. Introduction

Adolescence is a critical period marked by physical, psychological, and social changes [1]. This period represents a time of opportunity and changes but also a time of vulnerability in which adolescents might experiment with a wide range of risky behaviors, including substance use and sexual behaviors [2,3]. Risky sexual behaviors (RSB), defined as behaviors leading to sexually transmitted diseases and unintended pregnancies [4], are common correlates of emotional problems in adolescence and play an important role in the development of mental health disturbances, as defined by the presence of symptoms, signs, or abnormal traits of psychopathology [5,6,7]

Research has shown that some mental health disturbances [6,8,9] may increase vulnerability to risky sexual behaviors, unintended pregnancies, and sexually transmitted infections (STIs), including Human Immunodeficiency Virus/Acquired Immunodeficiency Syndrome (HIV/AIDS) [10]. According to the Centers for Disease Control and Prevention [11], people under 25 years old comprise around half of all new HIV cases, the majority of whom are infected through unprotected sexual intercourse. Among young people, epidemiological studies have shown that the incidence of mental health problems increases dramatically during adolescence, with around 25% of youths experiencing a depressive episode by the age of 18 [12,13]. However, the prevalence of mental health disturbances is much more pronounced among individuals involved in unprotected sex (sex without protection) and those with HIV. For example, a study examining the relationship between depression and risky sexual behaviors, including not using condoms, in adolescents found that students reporting depressive symptoms were three times more likely to practice risky sexual behaviors. Additionally, nearly 60% of participants surveyed, including those who displayed depression, reported that they did not use a condom during their last sexual intercourse, and 24.4% had not used condoms in the past 3 months [14].

Research has demonstrated that risky sexual behavior among adolescents differs by gender. It has been reported that men tend to use condoms less frequently than women [15,16,17] and have a greater number of sexual partners than women. Men also engage in sexual behavior at an earlier age, which is considered a risk factor for STIs and HIV [18]. A growing proportion of the literature has also found that self-identified sexual minority youth (such as lesbian, gay, and bisexual individuals) are also more likely than heterosexually-identifying adolescents to report high levels of anxiety, stress, and depression and experience risky sexual outcomes (sex while intoxicated, early intercourse debut, and STIs) [19,20,21]. It has been suggested that discrimination and stigma related to one’s minority status may impact mental health status and put them at risk of vulnerability to risky sexual behaviors [22,23]. In addition, a recent body of evidence suggests that adolescents who experience poorer mental health and practice risky sexual behaviors are at higher risk of experiencing more adverse events, including partner violence victimization, defined as physical, psychological, and sexual assaults by an intimate partner [24,25,26,27]. Among these individuals, the risk of having STIs and HIV is 1.5 times more likely compared with those who have not been abused by their partners [25]. Furthermore, partner violence during adolescence may lead to the exacerbation of mental health disturbances that may increase self-destructive behavior (e.g., inconsistent condom use and multiple sexual partners) [28].

Understanding the association between mental health problems and risk factors associated with risky sexual behaviors is crucial to the development of effective strategies for prevention. However, most of the research on the relationship between mental health problems and not using condoms has been conducted in North America and Africa, and to the best of our knowledge, no such association has been examined in some European regions, such as Spain. Previous studies conducted in Spain have reported that the prevalence of HIV/AIDS is above the European Union’s average prevalence [29] and that condom use has declined in the past decades in Spanish adolescents [30]. Therefore, identifying risk factors associated with the inconsistent use of condoms in Spanish adolescents is important.

Furthermore, most studies to date have looked at the relationship between depression and risky sexual behaviors but have not controlled for or examined the mediating role of self-esteem. Self-esteem, defined as positive and negative attitudes toward oneself [31], has been widely recognized as a central aspect of psychological health [32]. Previous studies have reported the existence of an inverse association between self-esteem and symptoms of depression during adolescence [33]. It has been reported that low self-esteem may be an important mediator of the relationship between mental health problems and sexual risk-taking behaviors since lower levels of self-esteem are linked to higher levels of depression, inconsistent use of condoms, unwanted pregnancies, multiple risk sexual partners, and early sex initiation [7,34]. Previous studies have reported that individuals with lower self-esteem may experience more difficulties in proposing the use of safer sex practices because they are more susceptible to pressure to engage in risky sex [35]. In addition, studies examining the role of self-esteem and depression have reported that low self-esteem is a risk factor in the etiology of depression [36] that may, in turn, increase vulnerability to feeling worthless, incompetent, and inadequate [37].

In this study, we examined the impact of mental health disturbances and partner violence victimization on condom use in the past 12 months among young people in Spain. We also examined the impact of self-esteem as a mediator between mental health problems and not using condoms. We hypothesized that (i) mental health disturbances and partner violence victimization are associated with inconsistent use of condoms and that (ii) self-esteem mediates the relationship between mental health problems and not using condoms in the past 12 months.

## 2. Materials and Methods

### 2.1. Participants

The present study was a part of a large-scale cross-sectional study on drug abuse and risky behaviors conducted in Alicante, Spain. The study involved 831 participants randomly selected from 25 public or semi-private secondary schools in Alicante (Spain) who were part of a previous study on substance use among adolescents. Criteria for student participant selection included class attendance on the day of the survey, fluency in Spanish, and the ability to complete a self-administered questionnaire on a computer. For the current study, we excluded all participants who declared not having a history of sexual intercourse, defined as any kind of sex between two or more people. From the initial sample, 468 (56.3) individuals were excluded given that they reported not having any sexual intercourse in the past 12 months, 19 (2.3%) participants were not in attendance on the day of the survey, 9 (1.1%) participants refused to participate, 23 (2.7%) participants were excluded because of missing data for one or more of the variables, and 27 (3.2%) surveys were discarded because of inconsistency in responses when the same question was repeated several times. In total, 285 (34.3%) students who reported having any sexual intercourse were included in the analyses.

### 2.2. Procedure 

Participants completed the National School-Based Survey (ESTUDES) [38], a standardized national survey on risky behaviors among adolescents. Data were collected from September 2018 to June 2019. Details about the ESTUDES methodology and its data can be publicly accessed from the Spanish Health Ministry [38]. The ESTUDES survey includes 90 questions about sociodemographic information, leisure activities, drug use, and risky behaviors in Spanish adolescents. The survey is based on the application of the Global School-Based Student Health Survey (GSHS) of the World Health Organization (WHO), whose methodology uses a “cluster sampling design in two stages (schools were selected by probability to size sampling and random selection of classrooms with students from secondary school)” [39,40]. In the current study, participants completed the national ESTUDES survey individually and anonymously in their classrooms after being informed in a clear and concise manner about how to fill out the survey questions and the confidentiality of their responses. All participants, and legal tutors for those under 18 years old, signed an informed consent agreement in which the study’s aims, methods, and assessment procedures were clearly stated, in accordance with Royal Decree 1720/2007 on Personal Data Protection. Participants were informed that their participation in the study was voluntary and that they could abandon the study with no consequences. The survey took approximately 60 min to complete.

The study was approved by the University of Alicante, the Generalitat Valenciana for Education, Research, Culture, and Sport, Ayuntamiento de Alicante (AYTOALICANTE3-18I), and the Educational Directive Committee for schools involved in the study (UA2018-10-13).

### 2.3. Measures 

Participants reported their gender (men or women); nationality (Spain or other countries); age; parents’ education level (certificate of school completion); partner physical, psychological, or sexual violence victimization (no, yes, or I have never gone out with a girl or boy); socioeconomic status (low, medium, or high); and school performance (exceptional, notable, good, passing, or failing). We also included two single-item measures related to sexual behaviors in the past 12 months. We also collected data on depression, anxiety, stress, and level of self-esteem.

#### 2.3.1. Socioeconomic Status (SES)

Socioeconomic status (SES) was analyzed with the Family Affluence Scale-II (FAS) [41], a short scale that examines socioeconomic status using four questions examining the following family resources: owning a vehicle (0 = none 1 = one, 2 = two or more), having own bedroom (0 = no, 1 = yes), having the possibility of traveling away on holiday (0 = not at all, 1 = once, 2 = twice, 3 = more than twice), and owning computers at home (0 = none, 1 = one, 2 = two, 3 = more than two). This scale can be regarded as an indirect measure of household income or family wealth. The total FAS score ranges from 0 to 9. According to the classification set forth by Boyce et al., (2006) [42], we coded each participant’s responses onto a three-point ordinal scale: low affluence (low FAS, score = 0, 1, or 2), middle affluence (middle FAS, score = 3, 4, or 5) and high affluence (high FAS, score ≥ 6). The scale showed adequate internal reliability, with alpha values ranging from 0.67 to 0.81.

#### 2.3.2. Sexual Behaviors

Sexual behaviors in the past 12 months were evaluated with two single-item measures: (i) “In the past 12 months, have you had any sexual intercourse”; (ii) “In the past 12 months, have you used condoms in your sexual intercourses?” Each question about sexual intercourse in the past 12 months had a binary response (yes or no).

#### 2.3.3. Parents’ Education

Parents ‘education was evaluated with a question included in the ESTUDES survey: “what was the highest level of education that your mother and father completed?”. We classified each participant’s responses into one of five categories (I = less than primary school, II = primary school, III = secondary school, IV = university, V = I do not know).

#### 2.3.4. School Performance

School performance was evaluated with two independent variables: marks at school and repeated courses. Marks at school were assessed with a question included in the ESTUDES survey: “What academic marks do you usually obtain?”. Responses were divided according to a five-point ordinal scale: (9–10 = exceptional, 7–8 = notable, 6 = good, 5 = passing, 0–4 = failing). Repeated courses were evaluated with a question included in the ESTUDES survey: “Have you ever repeated a course?”. This variable was classified into three categories (no = no, 1 course = yes, one course, ≥2 courses = yes, two courses or more.

#### 2.3.5. RSES—Rosenberg Self-Esteem Scale

The RSES is a 10-item self-reported questionnaire that assesses global self-confidence by evaluating both positive and negative feelings about oneself [43]. The RSES comprises 10 items (e.g., “I feel that I have a number of good qualities”). Participants rated the items on a 4-point Likert scale ranging from 0 (strongly disagree) to 4 (strongly agree). Higher scores indicated higher self-esteem. We classified self-esteem into three levels: low self-esteem = 1 (with values from 0 to 13); medium self-esteem = 2 (with values from 14 to 26); high self-esteem = 3 (with values from 27 to 40) in line with research that suggests that self-esteem levels are more accurate than global scores to describe this trait and make comparisons between groups [44]. For the present study, we used the Spanish version, which demonstrated adequate psychometric properties, with an alpha value of 0.83 [45]. In the present study, the items demonstrated sufficient internal reliability (α = 0.85).

#### 2.3.6. DASS-21—Depression, Anxiety, and Stress

The DASS 21—the Depression, Anxiety, and Stress Scale test [46]—is a self-reported questionnaire consisting of 21 items that evaluates the severity of mental disorder symptoms associated with depression, anxiety, and stress. Participants were asked to score every item on a scale from 0 (did not apply to me at all) to 3 (applied to me very much). Sum scores were computed by adding the scores on the items per (sub-) scale and multiplying them by a factor of 2. Summed scores for the total DASS-21 total scale range from 0 to 120, and those for each of the subscales range from 0 to 42. Scores ≥60 (for DASS-total) and ≥21 (for subscales) are labeled as “high” or “severe”. The scale showed sufficient internal reliability for the current sample, with an alpha value of 0.91 for the total score, 0.81 for depression, 0.81 for anxiety, and 0.86 for stress.

### 2.4. Statistical Analyses

A descriptive analysis of all variables was performed, calculating the number and frequency for the qualitative variables and the minimum, maximum, mean, and standard deviation for the quantitative ones. The Chi-squared test was applied to analyze the associations between condom use and the qualitative variables, and the Mann-Whitney U-test was used for the quantitative variables after testing the normality hypothesis using the Kolmogorov–Smirnov test. The association between explanatory variables and the three categories of self-esteem was analyzed through the adjustment of a multinomial ordinal model. To analyze the factors associated with the non-use of condoms in the past 12 months, prevalence ratios (PR) were estimated using Poisson models with robust variance [47] with 95% confidence intervals (95% CIs). To evaluate a possible mediating effect of self-esteem, the unadjusted PR was estimated, and a model that did not include self-esteem was adjusted, as was an additional model adjusted for the self-esteem variable. The R v4.0.2 program was used for the analysis.

## 3. Results

### 3.1. Sample Description

Table 1 and Table 2 provide a summary of the demographic information of the respondents. The mean age of the participants was 16.4 years (standard deviation (SD) = 0.99, range = 14.50 to 19.28). Concerning the participants, 79.6% were Spanish, 50.5% were men, 7.4% had low socioeconomic status, 30.2% had failing grades, 62.8% had repeated at least one course, and 11.6% had been expelled from school because of behavioral problems. Regarding the history of victimization, 8.4% had been a victim of physical or sexual abuse by their partners, and 40.5% had been a victim of psychological abuse by their partners.

Participants reported low depression, anxiety, and stress scores, with an overall mean of 11.79 in the total score of the DASS-21 (Table 2). Furthermore, 61.8% of the participants showed low levels of self-esteem, and 34.4% stated that they had not used condoms during sex.

### 3.2. Associations between Condom Use, Mental Health Disturbances, and Partner Violence Victimization

The factors that were significantly associated with not using condoms were having been a victim of physical, sexual, or psychological abuse; having been expelled from school because of behavioral problems; presenting high levels of depression or stress; and showing low levels of self-esteem (Table 1 and Table 2). In the multivariate analysis (Table 3), self-esteem, depression, anxiety, and stress were independently associated with the non-use of condoms, adjusted for age and sex; having low self-esteem increased the risk of not using condoms more than having high levels of self-esteem. For each unit of increase in the score on the depression subscale, the probability of not using condoms increased by 19%. For each unit of increase for stress, the risk of not using condoms increased by 9%, and the risk increased by 19% for each unit of increase for anxiety. Stress was not significantly associated with self-esteem.

### 3.3. Mediating Effect of Self-Esteem

Table 3 shows that depression and anxiety were significantly and independently associated with self-esteem, after adjusting for age and gender, such that self-esteem could be a possible mediator between these two factors and condom use.

Table 4 shows the two models adjusted for the non-use of condoms: model I does not consider self-esteem, and model II includes self-esteem. The results showed that the self-esteem factor was independently associated with the non-use of condoms. The presence of self-esteem did not modify the effect between depression and anxiety with the non-use of condoms, so self-esteem was not identified as a mediating factor.

## 4. Discussion

This study examined the impact of mental health disturbances and partner violence victimization on condom use in the past 12 months among young people in Spain. We also examined the impact of self-esteem as a mediator between mental health problems and not using condoms.

Three major findings emerged from this study. First, one in three adolescents between 14 and 19 years old did not use condoms during sexual intercourse. Contrary to most studies on gender differences regarding risky sexual behavior among adolescents, which suggest that men tend to use condoms less frequently than women, our results did not find significant differences in the use of condoms by sex. However, the prevalence of the non-use of condoms was higher in women. These results were in line with some prior studies among Latino youths suggesting that men who want to use condoms are more likely to do so compared with women, particularly when verbal and nonverbal communication strategies are used to communicate and negotiate about safer sex [48]. In addition, a study on increased levels of risky sexual behaviors among adolescents in Spain found that compared with boys, girls tended to use condoms less, particularly when they were in a stable relationship [49]. Our results also suggest that being physically, psychologically, or sexually abused by a partner and having lower levels of self-esteem and higher levels of depression and stress were significantly associated with not using condoms. These findings are consistent with previous research showing that exposure to different forms of partner violence, including physical, psychological, and sexual abuse, is associated with the non-use of condoms among adolescents [24]. Some explanatory mechanisms may influence this relationship, particularly among those who have been exposed to different forms of partner violence. One hypothesized mechanism is that adolescents who are victims of partner violence do not feel confident in requesting or discussing condom use and safer sexual activity with their partners because of the perception of coercive responses from their partners [50,51]. Furthermore, abused individuals are less likely to attempt to exert authority over safe sex practices because of feelings of fear and partner dependence [52,53]. In addition, it has been reported that individuals who are victims of partner violence often prioritize the immediate risk of injury over the risk of negative consequences associated with the non-use of condoms [54]. Therefore, these mechanisms may serve as barriers to practicing safer sex among those who have a history of experiencing partner violence [51].

Interestingly, the levels of depression, anxiety, and stress in our study were significantly associated with the non-use of condoms. For each unit of increase in the depression and anxiety score, the probability of not using condoms increased by 19%, and for each unit of increase in the stress score, the risk of not using condoms increased by around 9%. The association between victimization, behavioral problems, and psychological symptoms and not using condoms found in the present study is robust, and it remained when age and gender were controlled. These results are in line with previous studies that have demonstrated that mental health disturbances, including depression, anxiety, and stress, may act as barriers to condom use [8,9,55].

Second, our results suggest that having been suspended or expelled from school because of behavioral problems increases the risk of the non-use of condoms among adolescents, adding new evidence for the association between behavioral problems and risky sexual behaviors in Spain. It has been reported that students who are expelled from school are involved in more unhealthy behaviors, including unprotected sex, substance use, and learning difficulties, which may have a negative impact on self-esteem and psychological well-being, as suggested by the current results [56,57].

Third, the results of the present study also showed that self-esteem was independently associated with not using condoms. Contrary to our hypothesis, self-esteem did not mediate the relationship between mental health problems and the non-use of condoms in adolescents. According to our results, having lower levels of self-esteem increased the risk of not using condoms during sexual intercourse. These results are in line with previous research suggesting that individuals with lower self-esteem may experience more difficulties in proposing the use of safe sex practices given they are more susceptible to pressure to engage in risky sex [35]. Additionally, it has been reported that adolescents with low self-esteem typically lack confidence in themselves; display more depressive symptoms; and are more likely to respond to stressful situations with unhealthy behaviors, such as unprotected sexual behaviors [58,59]. The results of this study support evidence that demonstrates that lower levels of self-esteem and higher levels of depression are reciprocally associated and may play an important role in the etiology of depression [36]. However, while the extended literature to date has confirmed the influence of self-esteem on depression, the relation between self-esteem and anxiety has only rarely been examined [60]. This study found that higher levels of anxiety were associated with higher levels of self-esteem. To the best of our knowledge, no previous studies have reported such an association. However, a meta-analysis of longitudinal studies on how low self-esteem predicts depression and anxiety reported that low self-esteem had stronger predictive effects on depression than on anxiety. It is possible that students with higher levels of self-esteem and higher anxiety felt more confident in themselves and their decisions and were thus less susceptible to peer pressure in stressful situations that generate high levels of anxiety.

The results found in this study have important implications for understanding the impact of symptoms of depression, anxiety, and stress on the prevalence of the non-use of condoms among adolescent students. These findings enhance the importance of considering psychological well-being when implementing sexual health interventions for adolescents in school settings [57,61]. Research has demonstrated that mental health disturbances emerge in adolescence and are essential mechanisms involved in condom use among adolescents. Therefore, it is important that intervention programs for young people provide health education initiatives aimed at increasing the knowledge of condom use, particularly among students who are more vulnerable to mental health disturbances and behavioral problems [62]. In addition, given that schools provide better educational opportunities for prosocial behaviors, it is crucial that the implementation of psychoeducational interventions focuses on promoting healthy behaviors and positive attitudes about sexuality and self-respect among adolescent students [63,64].

Despite the importance of these findings, this study has several limitations. First, the main limitation is that our sample was limited to adolescent students in Spain who are not representative of young people in general. Second, given that the current study was a part of a large-scale study about risky behaviors in adolescent students, some variables assessed in this study were derived from single-item measures, a common technique in epidemiological research. Future studies can be performed using validated multi-item measures of the construct by also including possible determinants of the non-use of condoms among adolescent students. Third, because of the use of self-reported measures of sexual behaviors, our data may be influenced by reporting biases or social desirability. Future research should evaluate the effectiveness and feasibility of culturally tailored interventions for Spanish adolescents that target some of the variables found in this study associated with adolescents’ risky sexual behaviors. Randomized clinical trials examining whether these interventions reduce risky sexual behaviors need to be conducted before there are any implications for policy adoption [65]. Fourth, unprotected sex was only analyzed taking into consideration condom use. Future studies should include the use of different contraception methods, such as the contraceptive pill, female condoms, contraceptive implants, vaginal rings, etc., to analyze the actual amount of sexual intercourse and the proportion that is unprotected. Fifth, although the National School-Based Survey (ESTUDES) [38] is a validated and widely used survey to assess sociodemographic information, leisure activities, drug use, and risky behaviors in Spanish adolescents, it does not capture specific information on either sexual orientation or type of sexual activity. Thus, this study focused on studying unprotected vaginal–penis sexual intercourse among adolescents, assuming the participants in our sample were heterosexual. Therefore, this might have led to underestimating the presence of other sexual orientations and participants with same-sex intercourse who are more likely to have oral sex and anal sex instead of vaginal–penis sex; these variables may have influenced our results. Future research should examine whether different sexual activities have a direct effect on condom use and whether there are differences in sexual orientation.

## 5. Conclusions

In conclusion, our results suggest that among the risk factors associated with not using condoms, partner violence; low levels of self-esteem; and high levels of depression, anxiety, and stress are significantly associated with unsafe sex in young people. We also demonstrated that being expelled from school because of behavioral problems increased the risk of not using condoms, adding new evidence for the association between behavioral problems and sexual risk behaviors in Spain. The findings from this study suggest that efforts to increase adolescents’ condom use should incorporate mental health strategies in order to help youths perceive the necessity of safe sex. In addition, future studies dealing with young people should provide health education initiatives aimed at increasing awareness of condom use, particularly among students who are more vulnerable to emotional distress and mental disturbances.

## Figures and Tables

**Table 1:** Condom use vs. no condom use among adolescent students.

		Total		Condom Use		No Condom Use			
		*n*	%	*n*	%	*n*	%	Chi^2^ (df)	*p*-Value *
Gender	Men	144	50.5%	100	69.4%	44	30.6%	1.8 (1)	0.169
	Women	141	49.5%	87	61.7%	54	38.3%		
Country	Other	58	20.4%	41	70.7%	17	29.3%	0.8 (1)	0.362
	Spain	227	79.6%	146	64.3%	81	35.7%		
SES	Low	21	7.4%	13	61.9%	8	38.1%	1.0 (2)	0.598
	Medium	136	47.7%	86	63.2%	50	36.8%		
	High	128	44.9%	88	68.8%	40	31.3%		
Mother’s education	Less than primary school	11	3.9%	5	45.5%	6	54.5%	6.7 (4)	0.240
	Primary school	24	8.4%	16	66.7%	8	33.3%		
	Secondary school	117	41.1%	74	60.6%	43	39.4%		
	University	68	23.9%	43	63.2%	25	36.8%		
	I don’t know	65	22.8%	49	75.4%	16	24.6%		
Father’s education	Less than primary school	13	4.6%	8	61.5%	5	38.5%	3.6 (4)	0.598
	Primary school	15	5.3%	8	53.3%	7	46.7%		
	Secondary school	109	38.2%	68	62.0%	41	38.0%		
	University	63	22.1%	41	65.1%	22	34.9%		
	I don’t know	85	29.8%	62	72.9%	23	27.1%		
Marks at school	Exceptional (9–10)	18	6.3%	16	88.9%	2	11.1%	6.9 (4)	0.138
	Notable (7–8)	80	28.1%	55	68.8%	25	31.3%		
	Good (6)	101	35.4%	64	63.4%	37	36.6%		
	Passing (5)	66	23.2%	38	57.6%	28	42.4%		
	Failing (0–4)	20	7.0%	14	70.0%	6	30.0%		
Repeated courses	No	106	37.2%	70	66.0%	36	34.0%	0.02 (2)	0.990
	Yes, one course	96	33.7%	63	65.6%	33	34.4%		
	Yes, two or more courses	83	29.1%	54	65.1%	29	34.9%		
Partner physical violence	No	225	78.9%	147	65.3%	78	34.7%	24.9 (2)	<0.001
	Yes	24	8.4%	7	29.2%	17	70.8%		
	I’ve never gone out with a boy/girl	36	12.6%	33	91.7%	3	8.3%		
Partner sexual violence	No	221	77.5%	136	61.5%	85	38.5%	15.0 (2)	0.001
	Yes	24	8.4%	14	58.3%	10	41.7%		
	I’ve never gone out with a boy/girl	40	14.0%	37	92.5%	3	7.5%		
Partner Psychological	No	214	75.1%	137	64.0%	77	36.0%	22.2 (2)	<0.001
violence	Yes	30	10.5%	12	40.0%	18	60.0%		
	I’ve never gone out with a boy/girl	41	14.4%	38	92.7%	3	7.3%		
Expelled from school	No	252	88.4%	180	71.4%	72	28.6%	32.6 (1)	<0.001
	Yes	33	11.6%	7	21.2%	26	78.8%		
Self-esteem (RSES)	Low self-esteem	176	61.8%	100	56.8%	76	43.2%	22.9 (2)	<0.001
	Moderate self-esteem	73	25.6%	52	71.2%	21	28.8%		
	High self-esteem	36	12.6%	35	97.2%	1	2.8%		

Chi2 (df): value of Chi-square (degrees of freedom); * Chi-squared test.

**Table 2:** Mean values of quantitative variables for condom use.

	Min	Max	Mean	SD	Condom Use	*n*	Mean	SD	U *	*p*-Value *
Age	14.50	19.28	16.39	0.99	No	187	16.41	1.01	9019.5	0.828
					Yes	98	16.37	0.96		
Depression	0.00	18.00	4.81	3.84	No	197	3.14	3.07	2245.0	<0.001
					Yes	88	8.00	3.06		
Anxiety	0.00	17.00	2.36	2.39	No	187	2.50	2.66	8819.9	0.597
					Yes	98	2.09	1.74		
Stress	0.00	18.00	4.62	3.81	No	186	3.54	3.65	4275	<0.001
					Yes	99	6.68	3.24		
DASS-21 Total	0.00	53.00	11.79	8.34	No	187	9.18	8.25	3623.5	<0.001
					Yes	98	16.78	5.91		

* Mann-Whitney *U*-test.

**Table 3:** Multinomial ordinal regression for self-esteem.

	Value	Std. Error	*t*-Value	*p*-Value
Gender—Girl	0.001	0.247	0.004	0.997
Age	0.130	0.124	1.046	0.296
Depression	−0.121	0.040	−3.012	0.003
Anxiety	0.154	0.064	2.411	0.016
Stress	−0.021	0.045	−0.460	0.646
Intercepts				
1|2	2.311	2.066	1.118	0.264
2|3	3.821	2.075	1.842	0.065

**Table 4:** Multivariate analysis of Poisson regression with robust variance for non-use of condoms.

		Unadjusted			Model I			Model II		
		PR	95% CI	*p*	PR	95% CI	*p*	PR	95% CI	*p*
Self-esteem	High self-esteem	1			-			1		
	Moderate self-esteem	10.36	(1.45–73.97)	0.020	-			6.21	(0.92–42.09)	0.061
	Low self-esteem	15.55	(2.23–108.2)	0.006	-			7.31	(1.09–49.14)	0.041
Gender	Men	1								
	Women	1.25	(0.91–1.71)	0.171	1.00	(0.76–1.32)	0.996	1.01	(0.77–1.32)	0.941
Country	Other country	1								
	Spain	1.22	(0.79–1.88)	0.377						
SES	Low	1								
	Medium	0.97	(0.54–1.74)	0.906						
	High	0.82	(0.45–1.50)	0.520						
Mother’s education	I	1								
	II	0.61	(0.28–1.34)	0.217						
	III	0.83	(0.43–1.61)	0.586						
	IV	0.61	(0.33–1.13)	0.119						
	V	0.67	(0.36–1.26)	0.215						
	NS/NC	0.45	(0.23–0.90)	0.023						
Father’s education	I	1								
	II	1.21	(0.51–2.91)	0.665						
	III	1.01	(0.45–2.25)	0.978						
	IV	0.96	(0.45–2.04)	0.919						
	V	0.91	(0.42–1.95)	0.805						
	NS/NC	0.70	(0.33–1.52)	0.371						
Marks at school	9–10	1								
	7–8	2.81	(0.73–10.81)	0.132						
	6	3.30	(0.87–12.49)	0.079						
	5	3.82	(1.00–14.53)	0.049						
	0–4	2.70	(0.62–11.72)	0.185						
Repeated a course	No	1								
	1 course	1.01	(0.69–1.48)	0.951						
	≥2 courses	1.03	(0.69–1.53)	0.888						
Partner physical violence victimization	No	1								
	Yes	1.04	(1.49–2.79)	<0.001						
	NP	0.24	(0.08–0.72)	0.011						
Partner sexual violence victimization	No	1								
	Yes	1.08	(0.66–1.79)	0.755						
	NP	0.20	(0.06–0.59)	0.004						
Partner psychological violence victimization	No	1								
	Yes	1.67	(1.18–2.35)	0.003						
	NP	0.20	(0.07–0.61)	0.005						
Being expelled from school	No	1								
	Yes	2.76	(2.12–3.59)	<0.001						
Depression	(0–42)	1.19	(1.14–1.25)	<0.001	1.20	(1.15–1.25)	<0.001	1.19	(1.13–1.23)	<0.001
Anxiety	(0–42)	0.95	(0.88–1.02)	0.132	0.80	(0.74–0.85)	<0.001	0.81	(0.76–0.87)	<0.001
Stress	(0–42)	1.13	(1.09–1.16)	<0.001	1.09	(1.03–1.16)	0.002	1.09	(1.03–1.15)	0.002
DASS-21 total	(0–120)	1.05	(1.03–1.08)	<0.001						
Age	(years)	0.97	(0.83–1.14)	0.720	1.00	(0.88–1.14)	0.974	1.01	(0.89–1.14)	0.934

Model I: model not including self-esteem. Model II: optimal model with self-esteem. PR: prevalence ratio.

## Data Availability

Not applicable.
